# Hypoxia-Associated Remodeling of the Arginine–Citrulline–Ornithine Axis in Parkinson’s Disease and Restless Legs Syndrome: A Targeted LC–MS/MS and HIF-1α Profiling Study

**DOI:** 10.3390/medicina62071312

**Published:** 2026-07-07

**Authors:** Seyma Dumur, Mohammad Mahdi Bagheri Asl, Demet Aygun, Hafize Boyaci, Dildar Konukoglu, Hafize Uzun

**Affiliations:** 1 Department of Medical Biochemistry, Faculty of Medicine, Istanbul Atlas University, 34408 Istanbul, Turkey; hafize.uzun@atlas.edu.tr; 2 Department of Neurosurgery, Medical University of South Carolina, 135 Cannon Street, Charleston, SC 29425, USA; mmbagheriasl@gmail.com; 3 Department of Neurology, Faculty of Medicine, Biruni University, 34295 Istanbul, Turkey; demet.aygunustel@biruni.edu.tr; 4 Fikret Biyal Central Biochemistry Laboratory, Istanbul University-Cerrahpasa, 34303 Istanbul, Turkey; hafize.boyaci@iuc.edu.tr; 5 Department of Medical Biochemistry, Cerrahpasa Faculty of Medicine, Istanbul University-Cerrahpasa, 34098 Istanbul, Turkey; dkonuk@yahoo.com

**Keywords:** Parkinson’s disease, restless legs syndrome, HIF-1α, arginine metabolism, citrulline, ornithine, metabolomics, LC–MS/MS

## Abstract

*Background and Objectives*: Hypoxia-inducible factor-1 alpha (HIF-1α) is a central regulator of cellular responses to hypoxia and has been implicated in the pathophysiology of several neurological disorders. Parkinson’s disease (PD) and restless legs syndrome (RLS) have both been associated with alterations in oxygen sensing, mitochondrial dysfunction, and disturbances in amino acid metabolism; however, the relationship between HIF-1α and amino acid metabolic pathways in these disorders remains incompletely understood. The present study investigated circulating HIF-1α concentrations and amino acid metabolite profiles in patients with PD and RLS. *Materials and Methods*: In this cross-sectional study, 55 participants were enrolled, including 30 healthy controls, 12 patients with PD, and 13 patients with RLS. Plasma HIF-1α concentrations were measured using an enzyme-linked immunosorbent assay, and amino acid metabolites were quantified by liquid chromatography–tandem mass spectrometry. Group comparisons were performed using non-parametric methods with FDR correction. Age- and sex-adjusted regression analyses, correlation analyses, and PCA were used to assess metabolic relationships and group discrimination. *Results*: Significant group differences were observed for HIF-1α and multiple amino acid metabolites. Compared with controls, both PD and RLS patients exhibited significantly higher concentrations of arginine, citrulline, homocitrulline, and HIF-1α, whereas ornithine concentrations were significantly lower. Arginine demonstrated the largest effect size among all biomarkers (ε^2^ = 0.713). HIF-1α concentrations showed a progressive increase across groups, with the highest levels observed in RLS. Correlation analyses revealed strong positive associations of HIF-1α with arginine, citrulline, and homocitrulline, and an inverse association with ornithine. These findings remained significant after adjustment for age and sex. PCA showed clear separation between controls and disease groups. *Conclusions*: PD and RLS are characterized by a shared metabolic signature involving elevated HIF-1α, increased arginine-pathway metabolites, and reduced ornithine concentrations. The detected associations between HIF-1α and metabolites of the arginine–citrulline–ornithine pathway suggest a potential link between hypoxia-related signaling and metabolic dysregulation in both disorders. These findings support further investigation of HIF-1α-associated metabolic pathways as potential biomarkers and therapeutic targets in neurodegenerative and movement disorders.

## 1. Introduction

Parkinson’s disease (PD) is the second most common neurodegenerative disorder and is pathologically characterized by dopaminergic neuronal loss in the substantia nigra pars compacta together with intracellular α-synuclein-containing Lewy pathology [1,2]. Although motor symptoms remain the clinical hallmark of PD, the disease is increasingly recognized as a multisystem disorder involving non-motor manifestations, autonomic dysfunction, sleep disturbance, neuroinflammation, oxidative stress, and systemic metabolic dysregulation [1,2,3,4]. Among these mechanisms, mitochondrial impairment and redox imbalance have received particular attention, as they are believed to contribute to neuronal vulnerability, impaired bioenergetics, and progressive neurodegeneration [3,4,5].

Restless legs syndrome (RLS), also known as Willis–Ekbom disease, is a common sensorimotor disorder characterized by an urge to move the legs, typically accompanied by unpleasant sensations that occur or worsen during rest and in the evening or night [6,7]. Although RLS is clinically distinct from PD, several biological features overlap between the two disorders, including dopaminergic dysfunction, altered iron homeostasis, mitochondrial stress, oxidative imbalance, and sleep-related neurophysiological disruption [6,7,8,9]. These shared mechanisms raise the possibility that PD and RLS may involve convergent adaptive responses to chronic metabolic and cellular stress, despite their different clinical phenotypes.

In addition to these well-established mechanisms, increasing evidence indicates that environmental exposures and systemic metabolic disturbances contribute to the pathogenesis and progression of PD. Environmental factors, including pesticide exposure, heavy metals, and other neurotoxins, have been implicated in increasing disease susceptibility in genetically predisposed individuals. More recently, metabolic dysfunction has emerged as an additional component of PD pathophysiology. In particular, alterations in glucose homeostasis, increased glycemic variability, and episodes of hypoglycemia have been associated with disease severity and may exacerbate neuronal vulnerability through oxidative stress, mitochondrial dysfunction, and impaired cellular energy metabolism. These findings further support the concept that systemic metabolic dysregulation is closely linked to the pathophysiology of Parkinson’s disease [10].

Hypoxia-inducible factor-1 alpha (HIF-1α) is a master regulator of cellular adaptation to oxygen deprivation and metabolic stress [11,12]. Under hypoxic conditions, HIF-1α stabilizes and activates transcriptional programs that regulate angiogenesis, glucose metabolism, mitochondrial function, redox balance, inflammation, and cell survival [11,12,13]. Importantly, HIF-1α can also be activated under non-hypoxic conditions by oxidative stress, inflammatory mediators, mitochondrial dysfunction, and altered cellular metabolism [12,13,14]. In the nervous system, HIF-dependent signaling has been implicated in neuronal adaptation, glial responses, ischemic injury, neuroinflammation, and neurodegenerative processes [14,15]. However, the systemic metabolic correlates of circulating HIF-1α in human neurological disorders remain insufficiently characterized.

Amino acid metabolism represents a critical interface between mitochondrial function, nitrogen handling, endothelial regulation, neurotransmission, and immune-metabolic adaptation. Within this context, the arginine–citrulline–ornithine axis occupies a central position [16,17]. Arginine serves as the main substrate for nitric oxide synthase, generating nitric oxide and citrulline, thereby linking amino acid metabolism to vascular tone, neurotransmission, oxidative stress, and inflammatory signaling [16,17,18]. Alternatively, arginine can be metabolized by arginase to ornithine and urea, connecting nitrogen metabolism to polyamine synthesis, cellular growth pathways, and mitochondrial regulation [17,18,19]. Thus, coordinated changes in arginine, citrulline, and ornithine may reflect broader shifts in nitric oxide bioavailability, urea-cycle-related metabolism, polyamine turnover, and cellular stress adaptation.

Recent metabolomic studies have increasingly highlighted amino acid and nitrogen-related pathways as biologically informative in neurological and neurodegenerative disorders [19,20,21]. In particular, metabolites linked to arginine metabolism, including citrulline, ornithine, and homocitrulline, may provide insight into oxidative stress, nitric oxide metabolism, endothelial dysfunction, inflammatory burden, and polyamine-related metabolic pathways [16,17,18,19,20,21]. Related amino acid metabolites, such as histidine, β-alanine, carnosine, 1-methylhistidine, and 3-methylhistidine, may additionally reflect antioxidant capacity, protein turnover, muscle-related metabolic status, and systemic responses to chronic disease stress [22,23]. More broadly, amino acid metabolic networks are increasingly recognized as central mediators of systemic metabolic adaptation in human disease [24]. These metabolites therefore constitute an integrated biochemical network rather than isolated analytes.

Despite increasing interest in hypoxia signaling and metabolomic remodeling, these two domains have largely been studied separately in PD and RLS. In particular, whether circulating HIF-1α concentrations are associated with coordinated remodeling of the arginine–citrulline–ornithine axis in these disorders remains unclear. Addressing this gap may be important for identifying shared metabolic signatures that transcend conventional diagnostic categories and for improving our understanding of hypoxia-linked biochemical adaptation in neurological disease.

Therefore, the present study aimed to investigate circulating HIF-1α concentrations and amino acid metabolite profiles in patients with Parkinson’s disease and restless legs syndrome compared with healthy controls. Particular emphasis was placed on metabolites involved in the arginine–citrulline–ornithine pathway, given their potential links to hypoxia-responsive signaling and neurodegenerative processes. We hypothesized that PD and RLS would exhibit a shared metabolic signature characterized by altered HIF-1α activity and dysregulation of arginine-related metabolic pathways.

## 2. Materials and Methods

### 2.1. Study Population

This observational cross-sectional study included 55 participants: 30 healthy controls, 12 patients diagnosed with Parkinson’s disease (PD), and 13 patients diagnosed with rest-less legs syndrome (RLS). Consecutive patients with PD or RLS attending the Department of Neurology at Istanbul Atlas University during the recruitment period were assessed for eligibility. Healthy controls were recruited from volunteers without a history of neurological disease, chronic inflammatory disease, or other major systemic illness and, whenever possible, were frequency-matched to the patient groups with respect to age and sex.

**Figure 1:**
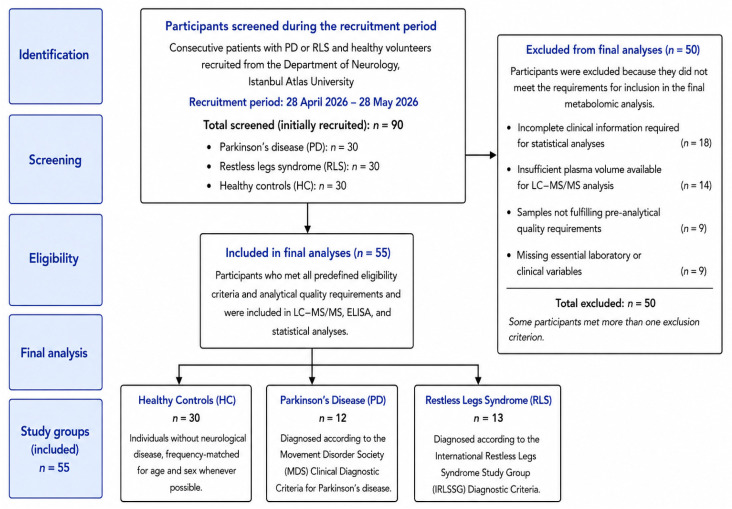
Flow diagram of participant recruitment, screening, exclusions, and final inclusion in the study cohort.

A total of 90 individuals (30 PD, 30 RLS, and 30 healthy controls) were initially recruited. After application of the predefined eligibility criteria and analytical quality requirements, 50 individuals were excluded because of incomplete clinical information, insufficient plasma volume for LC–MS/MS analysis, failure to meet pre-analytical sample quality criteria, or missing essential laboratory or clinical variables. Consequently, 55 participants were included in the final statistical analyses (Figure 1). All participants provided written informed consent before enrollment. The participant recruitment process is summarized in Figure 1.

Parkinson’s disease was diagnosed by experienced neurologists according to the Movement Disorder Society (MDS) Clinical Diagnostic Criteria. Diagnosis required the presence of parkinsonism, defined as bradykinesia in combination with either resting tremor or rigidity, together with supportive clinical criteria and the absence of absolute exclusion criteria or red flags incompatible with PD. Restless legs syndrome was diagnosed according to the International Restless Legs Syndrome Study Group (IRLSSG) diagnostic criteria, requiring fulfillment of all five essential diagnostic criteria: (i) an urge to move the legs usually accompanied by unpleasant sensations, (ii) symptom onset or worsening during rest or inactivity, (iii) partial or complete relief by movement, (iv) worsening during the evening or night, and (v) symptoms not solely attributable to another medical or behavioral condition.

Exclusion criteria included active infection, malignancy, chronic inflammatory disease, severe renal or hepatic dysfunction, autoimmune disorders, and the use of medications unrelated to the treatment of PD or RLS that are known to substantially alter amino acid metabolism. Standard disease-specific therapies were permitted and documented at enrollment. Demographic and clinical information, including disease duration, detailed medication history (including dopaminergic therapy and other regularly prescribed medications), ferritin concentrations, and neurological severity scores, was recorded at enrollment. Owing to the limited sample size and the heterogeneity of treatment regimens, medication exposure was summarized descriptively but was not included as a covariate in the statistical analyses. The study was conducted in accordance with the Declaration of Helsinki and was approved by the Non-Interventional Clinical Research Ethics Committee of Istanbul Atlas University (Decision No. 04/55, dated 27 April 2026; document number E-22686390-050.99-98564). Written informed consent was obtained from all participants prior to inclusion.

### 2.2. Blood Collection and Sample Preparation

Following an overnight fast, peripheral venous blood samples were collected between 08:00 and 10:00 a.m. into EDTA-containing tubes. Samples were centrifuged promptly after collection at 5000 rpm for 5 min, and plasma aliquots were separated and stored at −80 °C until analysis. Repeated freeze–thaw cycles were avoided. Immediately after centrifugation, plasma samples were visually inspected for evidence of hemolysis. Samples demonstrating visible hemolysis were excluded prior to biochemical analysis. Ferritin concentrations were determined in all participants with PD and RLS as part of the routine clinical assessment. Iron deficiency was not considered an exclusion criterion because altered iron homeostasis represents an established component of the pathophysiology of RLS.

### 2.3. Quantification of Plasma Amino Acid Metabolites by LC–MS/MS

Targeted amino acid profiling was performed using the ClinMass^®^ Plasma Amino Acids LC–MS/MS Complete Kit (MS14000; RECIPE Chemicals + Instruments GmbH, Munich, Germany). Chromatographic separation was achieved on an Accucore™ HILIC analytical column (150 × 3 mm; Thermo Fisher Scientific, Waltham, MA, USA). The mobile phase consisted of acetonitrile and 100 mM ammonium formate (90:10, *v*/*v*; pH 3.0). Prior to sample analysis, the LC–MS/MS system was equilibrated using a mobile phase composition of 88:12 (Mobile Phase A Phase B) at a flow rate of 0.6 mL/min. Although the ClinMass^®^ amino acid panel quantifies a broad range of amino acids, the present study primarily focused on metabolites associated with the arginine–citrulline–ornithine pathway because of their established roles in nitric oxide metabolism, hypoxia signaling, mitochondrial function, endothelial biology, and neuroinflammation. Histidine, 1-methylhistidine, and 3-methylhistidine were additionally evaluated as exploratory biomarkers reflecting antioxidant capacity, protein turnover, and systemic metabolic adaptation in chronic neurological disorders.

Detection was performed using electrospray ionization tandem mass spectrometry (ESI–MS/MS) operated in multiple reaction monitoring (MRM) mode. The principal analytes investigated in the present study were monitored using the following precursor/product ion transitions: arginine (*m*/*z* 175 → 70.3), histidine (*m*/*z* 156 → 110.2), 1-methylhistidine (*m*/*z* 170.1 → 126.2), and 3-methylhistidine (*m*/*z* 170.1 → 124.1), all acquired in positive ionization mode (ESI+).

Quantification was based on isotope-labelled internal standards, including arginine-13C6 (*m*/*z* 185 → 77.3), histidine-13C6,15N3 (*m*/*z* 156 → 110.2), and lysine-13C6,15N2 (*m*/*z* 155.2 → 87.3). Retention times were 11.3 min for arginine, 14.3 min for histidine, 15.9 min for 1-methylhistidine, and 16.1 min for 3-methylhistidine.

Calibration was performed using a ClinCal^®^ six-level plasma calibrator set (RECIPE Chemicals + Instruments GmbH, Munich, Germany) (Levels 0–5; order no. MS14013). Internal quality assurance was monitored using ClinChek^®^ Plasma Controls (RECIPE Chemicals + Instruments GmbH, Munich, Germany) (Levels I and II; order no. MS14082), and freshly prepared quality-control samples were included in each analytical batch. Matrix blank injections were performed at the beginning of each analytical sequence to ensure system stability and reproducibility.

The analytical method showed excellent performance characteristics. Linearity ranges were 5–600 µmol/L for arginine, 10–500 µmol/L for histidine, and 5–500 µmol/L for 1-methylhistidine. Limits of detection (LOD) ranged from 0.5 to 3.4 µmol/L and limits of quantification (LOQ) ranged from 1.2 to 9.8 µmol/L for the studied metabolites. Analytical precision was high, with intra-assay and inter-assay coefficients of variation below 6.2% and 5.5%, respectively. Method validation, calibration procedures, and quality-control assessment were performed according to the manufacturer’s recommendations.

All plasma samples were analyzed in a single analytical batch. Internal quality-control samples were included throughout the analytical sequence according to the manufacturer’s recommendations to monitor analytical performance and batch stability. All measured analyte concentrations fell within the validated analytical range of the assay. No samples required dilution or repeat measurement because of concentrations outside the calibration range. Consequently, no samples were excluded from the analysis because of concentrations falling outside the validated analytical measurement range.

### 2.4. Measurement of Circulating HIF-1α

Circulating plasma HIF-1α concentrations were measured using a commercially available sandwich enzyme-linked immunosorbent assay (ELISA) kit (Elabscience, Houston, TX, USA) according to the manufacturer’s instructions. HIF-1α concentrations were determined using the Human HIF-1α ELISA Kit (Catalog No. E-EL-H6066), which has a reported analytical sensitivity of 37.5 pg/mL and a detection range of 62.5–4000 pg/mL. Absorbance was measured at 450 nm, and concentrations were calculated from standard calibration curves. HIF-1α concentrations were expressed as pg/mL.

### 2.5. Statistical Analysis

Statistical analyses were performed using R software (version 4.5.3; R Foundation for Statistical Computing, Vienna, Austria).

Continuous variables were summarized as medians and interquartile ranges (IQRs), whereas categorical variables were reported as frequencies and percentages. Normality assumptions were assessed using graphical inspection and the Shapiro–Wilk test.

Group comparisons for continuous variables were performed using the Kruskal–Wallis test. When global significance was detected, pairwise comparisons were conducted using Dunn’s test. Hodges–Lehmann estimates and corresponding 95% confidence intervals were calculated for pairwise effect estimation.

Categorical variables were compared using Fisher’s exact test. To account for multiple testing, false discovery rate (FDR) correction was applied using the Benjamini–Hochberg procedure.

Sensitivity analyses were performed using log-transformed biomarker concentrations. Multivariable linear regression models adjusted for age and sex were constructed to evaluate independent associations between study groups and metabolite concentrations.

Relationships among biomarkers were assessed using Spearman rank correlation coefficients. Correlation *p*-values were corrected for multiple comparisons using the Benjamini–Hochberg procedure.

Exploratory principal component analysis (PCA) was performed using scaled metabolite concentrations to evaluate global metabolic patterns and group separation.

A two-sided *p*-value < 0.05 was considered statistically significant.

Given the exploratory nature of the study and the relatively limited sample size, effect-size estimates and confidence intervals were interpreted alongside *p*-values to provide a more comprehensive assessment of the identified associations.

## 3. Results

### 3.1. Demographic and Clinical Characteristics

The study included 55 participants comprising 30 healthy controls, 12 patients with Parkinson’s disease (PD), and 13 patients with restless legs syndrome (RLS). Demographic and clinical characteristics are summarized in Table 1.

The median age of the overall cohort was 52 years (IQR: 37–59 years). Median age was 47 years (IQR: 34–55 years) in controls, 56.5 years (IQR: 54.5–65 years) in the PD group, and 53 years (IQR: 37–59 years) in the RLS group. Age differed significantly among groups (Kruskal–Wallis H = 10.63, df = 2, *p* = 0.005, FDR-adjusted *p* = 0.006, ε^2^ = 0.166). Pairwise comparisons demonstrated a significant difference between controls and PD patients (Hodges–Lehmann estimate: −15 years; 95% CI: −23 to −6 years; FDR-adjusted *p* = 0.003), whereas the remaining pairwise comparisons were not significant.

Sex distribution did not differ significantly across groups (*p* = 0.382, FDR-adjusted *p* = 0.382). Comorbidity status differed significantly among groups (Fisher’s exact *p* = 1.00 × 10^−4^, FDR-adjusted *p* = 2.00 × 10^−4^), with hypertension and diabetes mellitus observed exclusively in the PD group.

Disease-specific characteristics were summarized descriptively because they were not applicable to controls. Median disease duration was 12.5 years (IQR: 10–14 years) in PD and 4 years (IQR: 4–6 years) in RLS. In the PD group, the median Hoehn and Yahr stage was 2.5 (IQR: 2–3), the median UPDRS Part III score was 24.5 (IQR: 19–33), and the median levodopa-equivalent daily dose was 665 mg/day (IQR: 585–755 mg/day). Median ferritin concentrations were 47 ng/mL (IQR: 35.5–53.5 ng/mL) in PD and 12 ng/mL (IQR: 9–13 ng/mL) in RLS.

### 3.2. Group Differences in Circulating HIF-1α and Amino Acid Metabolites

Several measured biomarkers differed significantly among the three groups following FDR correction. Median biomarker concentrations and global comparison statistics are presented in Table 2.

**Figure 2:**
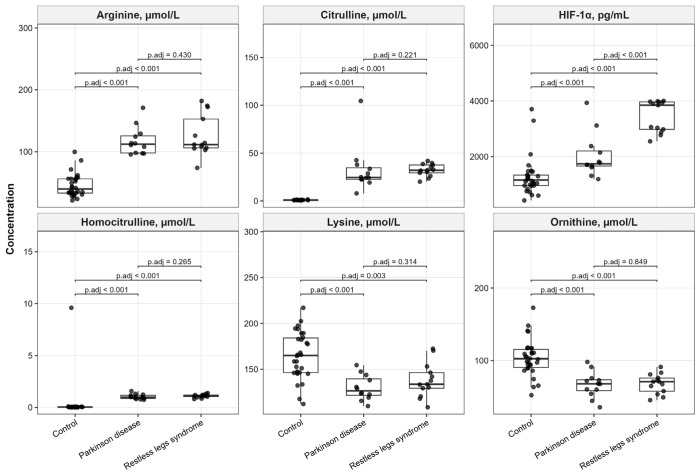
Distribution of key biomarkers across control, Parkinson’s disease, and restless legs syndrome groups. Boxplots show concentrations of arginine, citrulline, HIF-1α, homocitrulline, lysine, and ornithine in controls, Parkinson’s disease (PD), and restless legs syndrome (RLS) participants. Boxes represent the interquartile range (IQR), horizontal lines indicate medians, whiskers extend to 1.5 × IQR, and individual points represent participant values. Arginine demonstrated the largest overall group difference, with significantly higher concentrations in both PD and RLS compared with controls, while no significant difference was observed between PD and RLS.

Arginine exhibited the largest overall group difference (H = 39.07, df = 2, *p* = 3.29 × 10^−9^, FDR-adjusted *p* = 4.28 × 10^−8^, ε^2^ = 0.713). Median arginine concentrations were 39.72 µmol/L (IQR: 33.21–56.43 µmol/L) in controls, 112.03 µmol/L (IQR: 97.87–126.55 µmol/L) in PD, and 111.52 µmol/L (IQR: 106.14–152.73 µmol/L) in RLS. Pairwise analyses demonstrated significantly higher arginine concentrations in both PD (Hodges–Lehmann estimate: −69.58 µmol/L; 95% CI: −81.04 to −58.60 µmol/L; FDR-adjusted *p* = 2.00 × 10^−6^) and RLS (estimate: −75.69 µmol/L; 95% CI: −92.46 to −62.27 µmol/L; FDR-adjusted *p* = 1.00 × 10^−6^) compared with controls, whereas no significant difference was observed between PD and RLS (Figure 2; Figure S2).

Homocitrulline also differed significantly among groups (H = 35.28, df = 2, *p* = 2.18 × 10^−8^, FDR-adjusted *p* = 1.42 × 10^−7^, ε^2^ = 0.640). Median concentrations were 0.04 µmol/L (IQR: 0.03–0.05 µmol/L) in controls, 0.98 µmol/L (IQR: 0.86–1.19 µmol/L) in PD, and 1.10 µmol/L (IQR: 1.05–1.19 µmol/L) in RLS. Both disease groups exhibited significantly higher concentrations than controls, whereas no significant difference was observed between PD and RLS.

Citrulline measurements were available for 40 participants. Citrulline differed significantly across groups (H = 28.06, df = 2, *p* = 8.06 × 10^−7^, FDR-adjusted *p* = 2.62 × 10^−6^, ε^2^ = 0.704). Median concentrations were 0.88 µmol/L (IQR: 0.78–1.04 µmol/L) in controls, 24.67 µmol/L (IQR: 22.52–35.81 µmol/L) in PD, and 32.22 µmol/L (IQR: 29.57–37.67 µmol/L) in RLS. Pairwise analyses demonstrated significantly higher concentrations in both disease groups compared with controls, with no significant difference between PD and RLS.

Circulating HIF-1α concentrations differed significantly among groups (H = 32.67, df = 2, *p* = 8.03 × 10^−8^, FDR-adjusted *p* = 3.48 × 10^−7^, ε^2^ = 0.590). Median HIF-1α concentrations were 1157.17 pg/mL (IQR: 955.78–1349.62 pg/mL) in controls, 1743.96 pg/mL (IQR: 1648.68–2260.00 pg/mL) in PD, and 3845.85 pg/mL (IQR: 2972.26–3964.72 pg/mL) in RLS. Significant differences were observed between PD and controls, between RLS and controls, and between RLS and PD. The largest increase was observed in the RLS group (Figure 2).

Ornithine demonstrated an opposite pattern (H = 25.54, df = 2, *p* = 2.84 × 10^−6^, FDR-adjusted *p* = 6.82 × 10^−6^, ε^2^ = 0.453). Median concentrations were 102.60 µmol/L (IQR: 89.60–117.18 µmol/L) in controls, 68.21 µmol/L (IQR: 57.50–74.50 µmol/L) in PD, and 71.22 µmol/L (IQR: 58.24–76.12 µmol/L) in RLS. Both disease groups exhibited significantly lower concentrations than controls, whereas no significant difference was observed between PD and RLS.

**Figure 3:**
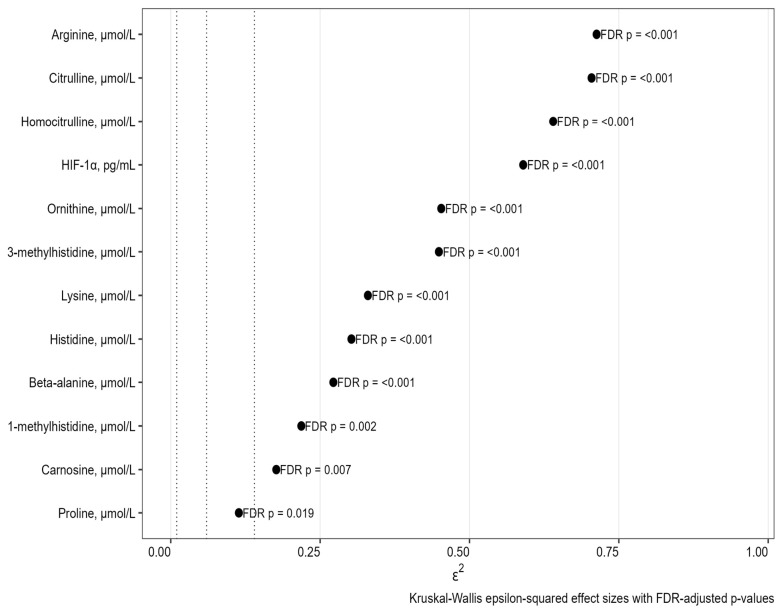
Effect sizes of global biomarker differences across study groups. Kruskal–Wallis epsilon-squared (ε^2^) effect sizes are shown for biomarkers demonstrating significant between-group differences. Points represent ε^2^ values, and adjacent labels indicate FDR-adjusted *p*-values. Vertical dashed lines denote conventional thresholds for small (ε^2^ = 0.01), medium (ε^2^ = 0.08), and large (ε^2^ = 0.26) effect sizes. Arginine, citrulline, homocitrulline, and HIF-1α exhibited the largest effects.

Additional biomarkers demonstrating significant global differences after FDR correction included 3-methylhistidine, lysine, histidine, β-alanine, 1-methylhistidine, carnosine, and proline (Figure 3; Figure S1).

### 3.3. Sensitivity Analyses

Sensitivity analyses using log-transformed biomarker concentrations yielded findings consistent with the primary non-parametric analyses. All biomarkers identified as significant in the primary analyses remained significant after FDR correction (Table S1). Additionally, age- and sex-adjusted regression analyses confirmed the observed associations (Table S2), supporting the robustness of the results.

### 3.4. Age- and Sex-Adjusted Analyses

Multivariable linear regression models adjusted for age and sex demonstrated that the observed biomarker differences remained significant after controlling for demographic variables.

Compared with controls, PD was independently associated with increased homocitrulline, citrulline, arginine, histidine, and HIF-1α concentrations, together with decreased ornithine, lysine, 1-methylhistidine, and 3-methylhistidine concentrations. The strongest positive associations were observed for citrulline, arginine, homocitrulline, and HIF-1α, whereas ornithine demonstrated a robust inverse association in both disease groups.

Similarly, RLS was independently associated with increased β-alanine, proline, homocitrulline, citrulline, arginine, histidine, and HIF-1α concentrations, as well as decreased ornithine, lysine, 1-methylhistidine, and 3-methylhistidine concentrations.

These findings indicate that the observed metabolic alterations were not attributable solely to differences in age or sex.

### 3.5. Correlation and Principal Component Analyses

**Figure 4:**
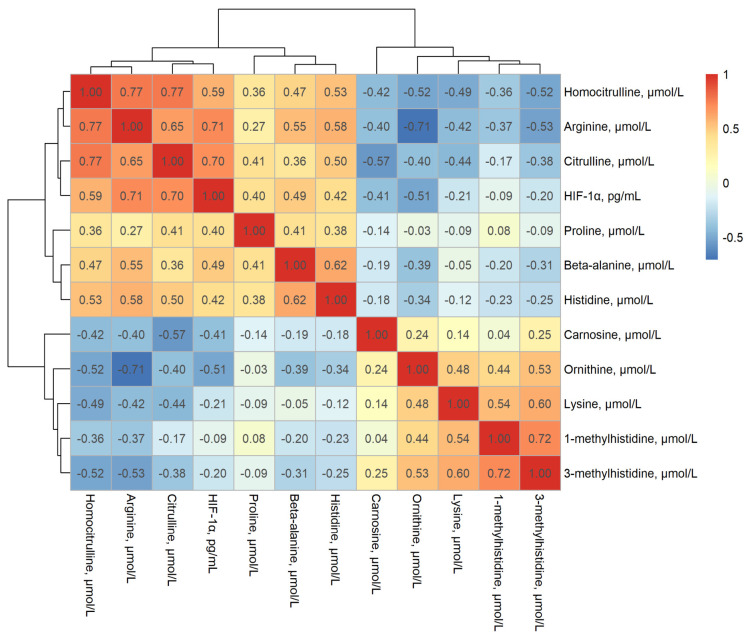
Spearman correlation heatmap of HIF-1α and amino acid biomarkers. Heatmap showing pairwise Spearman rank correlation coefficients (ρ) among HIF-1α and measured amino acid biomarkers. Color intensity indicates the strength and direction of correlations, with warmer colors representing positive associations and cooler colors representing negative associations. Strong positive correlations were observed between HIF-1α and metabolites within the arginine–citrulline–ornithine pathway.

Spearman correlation analysis identified strong associations between HIF-1α and metabolites involved in the arginine–citrulline–ornithine pathway (Figure 4).

Arginine demonstrated strong positive correlations with homocitrulline (ρ = 0.774), HIF-1α (ρ = 0.714), citrulline (ρ = 0.651), β-alanine (ρ = 0.553), and histidine (ρ = 0.583), while exhibiting inverse correlations with ornithine (ρ = −0.709) and 3-methylhistidine (ρ = −0.528). Citrulline was positively correlated with both homocitrulline (ρ = 0.765) and HIF-1α (ρ = 0.698). HIF-1α was positively correlated with homocitrulline (ρ = 0.588) and negatively correlated with ornithine (ρ = −0.513).

Among all observed correlations, the strongest biologically relevant association was identified between HIF-1α and arginine (ρ = 0.714), accompanied by a strong inverse association between HIF-1α and ornithine (ρ = −0.513) (Table S3).

**Figure 5:**
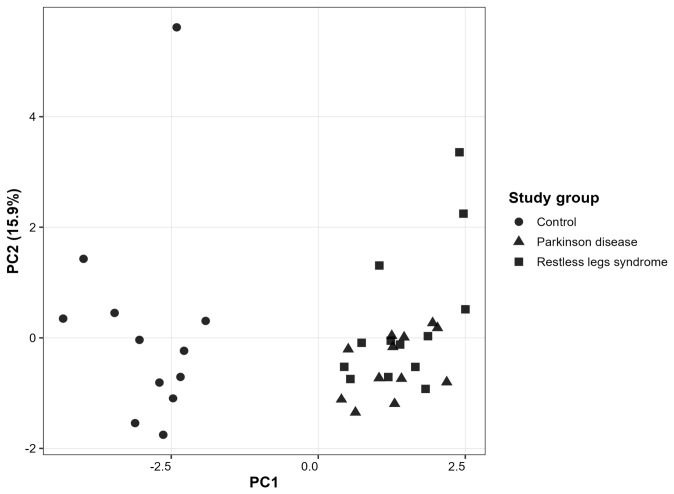
Principal component analysis of biomarker profiles across study groups. Principal component analysis (PCA) score plot based on measured biomarker concentrations. Principal component 1 (PC1) explained 38.1% of the total variance and principal component 2 (PC2) explained 15.9%, accounting for 54.0% of the overall variance combined. Participant scores demonstrated separation between controls and disease groups, while Parkinson’s disease (PD) and restless legs syndrome (RLS) showed partial overlap. Biomarkers contributing most strongly to group discrimination included HIF-1α, arginine, citrulline, homocitrulline, and ornithine.

Exploratory principal component analysis demonstrated that PC1 explained 38.1% of total variance and PC2 explained 15.9%, with the first two principal components accounting for 54.0% of total biomarker variance (Figure 5). Visualization of participant scores demonstrated separation between controls and disease groups, whereas PD and RLS exhibited partial overlap. Loading patterns indicated that HIF-1α, arginine, citrulline, homocitrulline, and ornithine were the major contributors to group discrimination.

## 4. Discussion

The present study investigated circulating HIF-1α concentrations together with targeted amino acid metabolite profiles in patients with Parkinson’s disease (PD) and restless legs syndrome (RLS). The principal findings were threefold. First, both PD and RLS were characterized by significantly elevated circulating HIF-1α concentrations compared with healthy controls, with the highest levels observed in RLS. Second, substantial alterations were identified within the arginine–citrulline–ornithine metabolic pathway, including increased arginine, citrulline, and homocitrulline concentrations together with reduced ornithine concentrations. Third, strong associations were observed between HIF-1α and metabolites of this pathway, and these relationships remained significant after adjustment for age and sex. Collectively, these findings suggest that hypoxia-responsive signaling and amino acid metabolic remodeling may represent shared biological features of PD and RLS.

To our knowledge, this is among the first studies to simultaneously evaluate circulating HIF-1α and targeted arginine-pathway metabolites in both PD and RLS. By integrating HIF-1α measurements with targeted LC–MS/MS-based metabolite profiling, the present study provides novel evidence that hypoxia-related signaling is closely linked to coordinated metabolic alterations in these neurologic disorders.

Increasing evidence indicates that dysregulated oxygen sensing, mitochondrial dysfunction, oxidative stress, and metabolic adaptation contribute to the pathophysiology of both neurodegenerative and movement disorders [3,4,5,9,14,15]. HIF-1α is a master regulator of cellular responses to hypoxic and metabolic stress and regulates numerous pathways involved in angiogenesis, mitochondrial function, glucose metabolism, inflammation, and cell survival [11,12,25,26]. Experimental and clinical studies have implicated HIF-dependent signaling in neurodegeneration, ischemic injury, and adaptive neuronal responses to cellular stress [14,15]. The elevated circulating HIF-1α concentrations observed in both disease groups are therefore consistent with the hypothesis that chronic activation of hypoxia-responsive pathways may occur in PD and RLS.

A particularly notable finding was the marked elevation of HIF-1α in the RLS group. Iron deficiency is a well-recognized feature of RLS and has been proposed as a key contributor to disease pathophysiology [8,27]. Experimental evidence indicates that iron availability influences HIF signaling through regulation of prolyl hydroxylases responsible for HIF-1α degradation [25,26]. The substantially lower ferritin concentrations observed in our RLS cohort support this concept and may partly explain the pronounced increase in circulating HIF-1α. Although causality cannot be established in this cross-sectional study, the coexistence of reduced ferritin and elevated HIF-1α is compatible with the growing view that altered hypoxia-related signaling contributes to RLS pathophysiology [8,9,27].

The most striking metabolic alterations involved the arginine–citrulline–ornithine pathway. Arginine demonstrated the largest effect size among all measured biomarkers and was accompanied by marked elevations in citrulline and homocitrulline. Arginine serves as a central substrate for nitric oxide synthesis and participates in pathways regulating vascular function, immune responses, oxidative stress, and cellular signaling [16,17,18]. Elevated citrulline may reflect altered nitric oxide metabolism and increased arginine turnover, whereas increased homocitrulline may indicate broader disturbances in nitrogen handling and amino acid metabolic adaptation [16,17,18,19,20,21,24]. The simultaneous elevation of these metabolites suggests coordinated remodeling of arginine metabolism rather than isolated biochemical abnormalities.

In contrast, ornithine concentrations were significantly reduced in both disease groups and demonstrated inverse correlations with HIF-1α and arginine. Ornithine occupies a central position at the intersection of the urea cycle and polyamine biosynthetic pathways, linking nitrogen disposal to cellular growth, mitochondrial regulation, and metabolic adaptation [16,17,18]. Reduced circulating ornithine may indicate altered metabolic flux through arginase-dependent pathways, potentially favoring arginine utilization within alternative pathways associated with cellular stress adaptation. The reciprocal pattern of increased arginine and decreased ornithine observed in both PD and RLS further supports the existence of pathway-level metabolic reorganization.

The correlation analyses provide additional support for this interpretation. Strong positive correlations were observed between HIF-1α and arginine, citrulline, and homocitrulline, whereas ornithine demonstrated a significant inverse association with HIF-1α. These relationships are biologically plausible given the recognized interactions among oxygen sensing, nitric oxide metabolism, mitochondrial regulation, and amino acid homeostasis [11,12,13,14,15,16,17,18,24,25]. The persistence of these associations after adjustment for age and sex strengthens the robustness of the findings.

The exploratory principal component analysis further supported the coordinated nature of these metabolic alterations. Clear separation between healthy controls and the disease groups was observed, whereas PD and RLS exhibited partial overlap, suggesting the presence of shared metabolic adaptations despite their distinct clinical phenotypes. HIF-1α, arginine, citrulline, homocitrulline, and ornithine contributed most strongly to group discrimination, reinforcing the central role of hypoxia-responsive signaling and remodeling of the arginine–citrulline–ornithine pathway in the metabolic phenotype identified in both disorders. Collectively, these findings indicate that the observed biomarker changes represent a coordinated metabolic network rather than isolated biochemical abnormalities.

Several limitations of this study should be acknowledged. First, the sample size was relatively modest, particularly within the disease groups, which may have limited the statistical power and generalizability of the findings. To reduce the risk of false-positive findings associated with a limited sample size, multiple complementary statistical approaches were applied, including false discovery rate (FDR) correction for multiple comparisons, effect-size estimation with 95% confidence intervals, sensitivity analyses using log-transformed biomarker concentrations, and multivariable linear regression models adjusted for age and sex. Nevertheless, despite these methodological safeguards, the identified associations should be interpreted with appropriate caution, particularly with respect to their generalizability to broader patient populations. Second, the cross-sectional design precludes causal inference and does not permit assessment of the temporal relationship between HIF-1α activation and metabolic alterations. Third, although detailed medication histories, including dopaminergic therapy, were recorded, medication exposure was not included as a covariate because of the limited sample size and the heterogeneity of treatment regimens. Consequently, residual confounding related to pharmacological treatment cannot be excluded. In addition, detailed information regarding comorbid medical conditions other than hypertension and diabetes mellitus was not systematically collected for all participants. Consequently, the potential influence of unmeasured comorbidities on circulating biomarker concentrations cannot be completely excluded.

Finally, all biomarker measurements were performed using peripheral blood samples rather than cerebrospinal fluid or brain tissue. Although circulating HIF-1α and amino acid metabolites may reflect systemic metabolic alterations associated with Parkinson’s disease and restless legs syndrome, peripheral biomarkers do not necessarily mirror biochemical processes occurring within the central nervous system because of tissue-specific metabolic regulation and the presence of the blood–brain barrier. Accordingly, the present findings should be interpreted as reflecting systemic metabolic changes rather than direct evidence of central nervous system pathology. Future studies incorporating cerebrospinal fluid analyses, neuroimaging biomarkers, or brain tissue investigations are warranted to further elucidate the relationship between peripheral and central metabolic alterations. Nevertheless, the use of targeted LC–MS/MS metabolomics, rigorous correction for multiple testing, effect-size estimation, and age- and sex-adjusted analyses strengthens the robustness of the present findings. Overall, the present findings should be regarded as hypothesis-generating and require confirmation in larger, independent prospective studies before definitive conclusions regarding disease-specific metabolic mechanisms can be drawn.

Future studies involving larger, multicenter cohorts, longitudinal follow-up, and integrated metabolomic and molecular approaches are warranted to clarify the mechanistic links between hypoxia-responsive signaling and amino acid metabolism. Furthermore, investigation of whether modulation of HIF-related pathways influences arginine metabolism and clinical outcomes may provide novel insights into the pathophysiology and therapeutic management of both PD and RLS.

## 5. Conclusions

In conclusion, patients with Parkinson’s disease and restless legs syndrome exhibited a shared biochemical profile characterized by elevated circulating HIF-1α concentrations, increased arginine, citrulline, and homocitrulline levels, and reduced ornithine concentrations. The strong associations between HIF-1α and metabolites of the arginine–citrulline–ornithine pathway suggest a potential interaction between hypoxia-responsive signaling and amino acid metabolic dysregulation. These findings identify a common metabolic signature across both disorders and support further investigation of HIF-1α-associated pathways as potential biomarkers and mechanistic contributors to neurodegenerative and movement disorders.

## Figures and Tables

**Table 1:** Demographic and clinical characteristics of the study population.

Variable	Control (n = 30)	Parkinson’s Disease (n = 12)	Restless Legs Syndrome (n = 13)	Overall (n = 55)	*p*-Value	FDR-Adjusted *p*-Value
Age, years	47.0 (34.0–55.0)	56.5 (54.5–65.0)	53.0 (37.0–59.0)	52.0 (37.0–59.0)	0.005	0.006
Female sex, n (%)	17 (56.7)	4 (33.3)	7 (53.8)	28 (50.9)	0.382	0.382
Male sex, n (%)	13 (43.3)	8 (66.7)	6 (46.2)	27 (49.1)	—	—
No comorbidity, n (%)	30 (100.0)	4 (33.3)	13 (100.0)	47 (85.5)	1.0 × 10^−4^	2.0 × 10^−4^
Hypertension, n (%)	0 (0.0)	5 (41.7)	0 (0.0)	5 (9.1)	—	—
Diabetes mellitus, n (%)	0 (0.0)	3 (25.0)	0 (0.0)	3 (5.5)	—	—

Data are presented as median (IQR) or n (%). Age was compared using the Kruskal–Wallis test; categorical variables were compared using Fisher’s exact test.

**Table 2:** Biomarker concentrations and global group comparisons.

Biomarker	Control	Parkinson’s Disease	Restless Legs Syndrome	H Statistic	FDR-Adjusted *p*-Value	Effect Size (ε^2^)
Arginine, µmol/L	39.72 (33.21–56.43)	112.03 (97.87–126.55)	111.52 (106.14–152.73)	39.07	4.28 × 10^−8^	0.713
Citrulline, µmol/L	0.88 (0.78–1.04)	24.67 (22.52–35.81)	32.22 (29.57–37.67)	28.06	2.62 × 10^−6^	0.704
Homocitrulline, µmol/L	0.04 (0.03–0.05)	0.98 (0.86–1.19)	1.10 (1.05–1.19)	35.28	1.42 × 10^−7^	0.640
HIF-1α, pg/mL	1157.17 (955.78–1349.62)	1743.96 (1648.68–2260.00)	3845.85 (2972.26–3964.72)	32.67	3.48 × 10^−7^	0.590
Ornithine, µmol/L	102.60 (89.60–117.18)	68.21 (57.50–74.50)	71.22 (58.24–76.12)	25.54	6.82 × 10^−6^	0.453
3-Methylhistidine, µmol/L	0.66 (0.57–0.78)	0.30 (0.24–0.43)	0.32 (0.29–0.43)	25.34	6.82 × 10^−6^	0.449
Lysine, µmol/L	165.19 (146.45–184.59)	126.61 (120.94–140.97)	133.71 (129.40–146.41)	19.16	1.30 × 10^−4^	0.330
Histidine, µmol/L	60.87 (56.61–64.46)	74.89 (65.35–83.71)	66.26 (61.57–71.79)	17.72	2.30 × 10^−4^	0.302
Beta-alanine, µmol/L	2.01 (1.35–2.34)	2.96 (2.42–4.07)	3.51 (2.40–3.84)	16.15	4.50 × 10^−4^	0.272
1-Methylhistidine, µmol/L	4.99 (3.58–12.53)	1.48 (0.93–2.83)	2.69 (1.23–8.47)	13.36	0.002	0.219
Carnosine, µmol/L	0.06 (0.03–0.08)	0.04 (0.01–0.06)	0.03 (0.02–0.04)	9.95	0.008	0.177
Proline, µmol/L	211.78 (175.13–239.45)	267.81 (173.51–326.34)	264.69 (226.26–334.86)	7.93	0.019	0.114

Data are presented as median (IQR). Global group differences were assessed using the Kruskal–Wallis test and adjusted for multiple comparisons using the Benjamini–Hochberg false discovery rate procedure. Citrulline measurements were available in 40 participants because of missing values.

## Data Availability

The raw data supporting the conclusions of this article will be made available by the authors on request.

## Supplementary Materials

The following supporting information can be downloaded at: https://www.mdpi.com/article/10.3390/medicina62071312/s1, Table S1: Sensitivity analysis of between-group differences using log-transformed biomarker concentrations. Table S2: Age- and sex-adjusted regression analyses of biomarker concentrations in Parkinson’s disease and restless legs syndrome relative to controls. Table S3: Significant Spearman correlations among HIF-1α and metabolites of the arginine–citrulline–ornithine pathway and related amino acids. Figure S1: Distribution of all biomarkers demonstrating significant between-group differences. Boxplots showing concentrations of 1-methylhistidine, 3-methylhistidine, arginine, β-alanine, carnosine, citrulline, HIF-1α, histidine, homocitrulline, lysine, ornithine, and proline across controls, Parkinson's disease, and restless legs syndrome participants. Boxes represent the interquartile range, center lines indicate medians, whiskers extend to 1.5 × IQR, and points represent individual observations. Figure S2: Pairwise Hodges–Lehmann estimates and 95% confidence intervals for key biomarkers. Forest plots showing median differences between controls, Parkinson's disease (PD), and restless legs syndrome (RLS) for selected biomarkers. Points represent Hodges–Lehmann estimates and horizontal bars indicate 95% confidence intervals. Values to the left of zero indicate lower concentrations in the first group listed, whereas values to the right indicate higher concentrations.

## Abbreviations

The following abbreviations are used in this manuscript:

PD	Parkinson’s Disease
RLS	Restless Legs Syndrome
HIF-1α	Hypoxia-Inducible Factor-1 Alpha
LC–MS/MS	Liquid Chromatography–Tandem Mass Spectrometry
MRM	Multiple Reaction Monitoring
ESI	Electrospray Ionization
PCA	Principal Component Analysis
UPDRS	Unified Parkinson’s Disease Rating Scale
LEDD	Levodopa-Equivalent Daily Dose
FDR	False Discovery Rate
IQR	Interquartile Range
CI	Confidence Interval
ELISA	Enzyme-Linked Immunosorbent Assay
NO	Nitric Oxide
LOD	Limit of Detection
LOQ	Limit of Quantification
CV	Coefficient of Variation
