# Supplementary material for: Hypoxia-Associated Remodeling of the Arginine–Citrulline–Ornithine Axis in Parkinson’s Disease and Restless Legs Syndrome: A Targeted LC–MS/MS and HIF-1α Profiling Study
Source: Medicina (Kaunas). 2026 Jul 7;62(7):1312. doi: 10.3390/medicina62071312 (PMC13413847; doi:10.3390/medicina62071312)
Supplement: Supplementary file 1 [file medicina-62-01312-s001.zip › medicina-4399479-supplementary.pdf]

Supplementary Materials:

Table S1. Sensitivity analysis of between-group differences using log-transformed biomarker concentrations.

Biomarker	FDR-adjusted p-value
Arginine	3.95×10^{-8}
Homocitrulline	1.31×10^{-7}
HIF-1α	3.21×10^{-7}
Citrulline	2.42×10^{-6}
Ornithine	6.30×10^{-6}
3-Methylhistidine	6.30×10^{-6}
Lysine	1.20×10^{-4}
Histidine	2.10×10^{-4}
β-Alanine	4.10×10^{-4}
1-Methylhistidine	0.002
Carnosine	0.008
Proline	0.019

Table S2. Age- and sex-adjusted regression analyses of biomarker concentrations in Parkinson's disease and restless legs syndrome relative to controls.

Biomarker	PD β (95% CI)	PD FDR-p	RLS β (95% CI)	RLS FDR-p
Arginine	1.018 (0.772–1.263)	8.63×10^{-11}	1.053 (0.843–1.263)	3.33×10^{-13}
Citrulline	2.717 (2.411–3.024)	7.12×10^{-19}	2.836 (2.565–3.108)	7.59×10^{-21}
Homocitrulline	0.693 (0.449–0.937)	1.59×10^{-6}	0.648 (0.438–0.857)	4.99×10^{-7}
HIF-1α	0.467 (0.172–0.762)	0.004	1.071 (0.818–1.324)	7.10×10^{-11}
Ornithine	−0.411 (−0.604 to −0.219)	1.30×10^{-4}	−0.396 (−0.561 to −0.231)	3.42×10^{-5}
Lysine	−0.214 (−0.327 to −0.102)	9.20×10^{-4}	−0.168 (−0.264 to −0.071)	0.002
Histidine	0.189 (0.083–0.295)	0.002	0.121 (0.031–0.212)	0.016
1-Methylhistidine	−0.935 (−1.590 to −0.281)	0.015	−0.642 (−1.203 to −0.080)	0.043
3-Methylhistidine	−0.274 (−0.397 to −0.150)	1.20×10^{-4}	−0.202 (−0.309 to −0.096)	5.90×10^{-4}
β-Alanine	NS	—	0.399 (0.175–0.623)	0.002
Proline	NS	—	0.257 (0.056–0.458)	0.033

Table S3. Significant Spearman correlations among HIF-1α and metabolites of the arginine–citrulline–ornithine pathway and related amino acids.

Variables	ρ	FDR-adjusted p-value
Arginine – Homocitrulline	0.774	2.80×10^{-10}
Arginine – HIF-1α	0.714	2.02×10^{-8}
Arginine – Citrulline	0.651	3.23×10^{-5}
Arginine – Histidine	0.583	2.02×10^{-5}
Arginine – β-Alanine	0.553	6.44×10^{-5}
Arginine – Ornithine	−0.709	2.35×10^{-8}
Arginine – 3-Methylhistidine	−0.528	1.50×10^{-4}
Citrulline – Homocitrulline	0.765	1.18×10^{-7}
Citrulline – HIF-1α	0.698	5.25×10^{-6}
HIF-1α – Homocitrulline	0.588	1.68×10^{-5}
HIF-1α – Ornithine	−0.513	2.20×10^{-4}

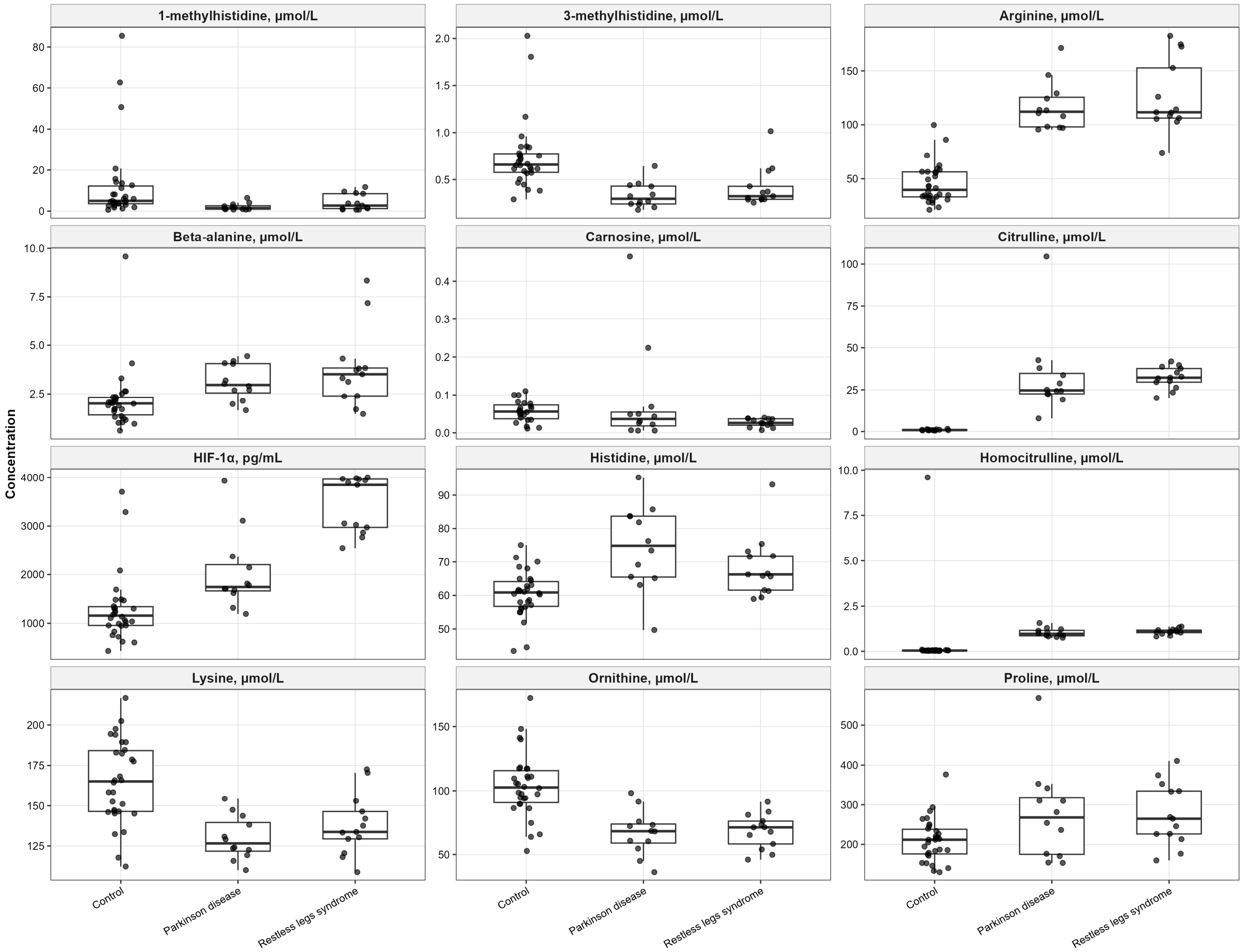

Figure S1. Distribution of all biomarkers demonstrating significant between-group differences. Boxplots showing concentrations of 1-methylhistidine, 3-methylhistidine, arginine, β-alanine, carnosine, citrulline, HIF-1α, histidine, homocitrulline, lysine, ornithine, and proline across controls, Parkinson's disease, and restless legs syndrome participants. Boxes represent the interquartile range, center lines indicate medians, whiskers extend to 1.5 × IQR, and points represent individual observations.

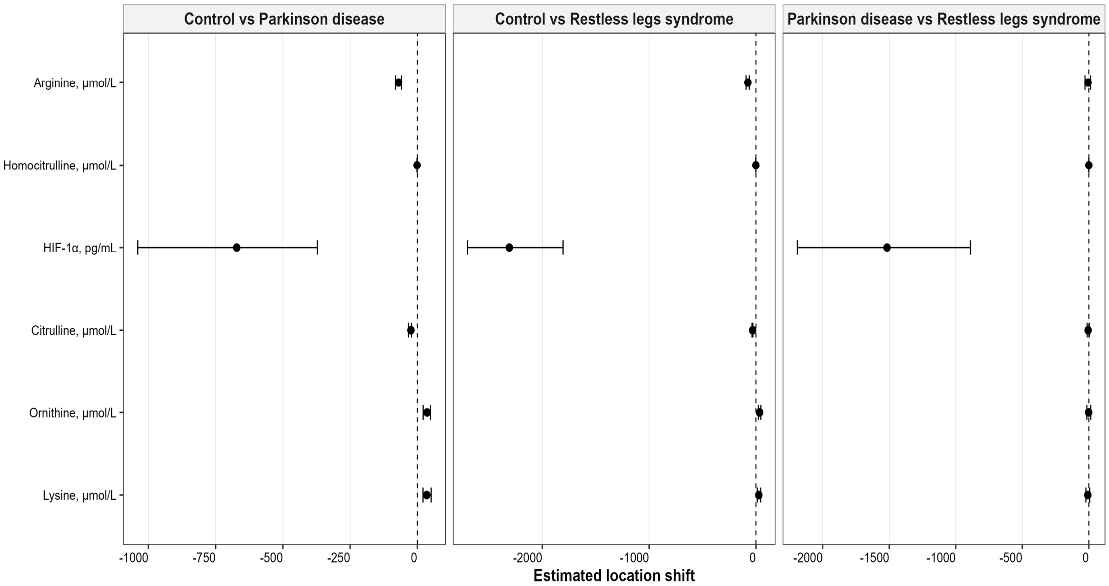

Figure S2. Pairwise Hodges–Lehmann estimates and 95% confidence intervals for key biomarkers. Forest plots showing median differences between controls, Parkinson's disease (PD), and restless legs syndrome (RLS) for selected biomarkers. Points represent Hodges–Lehmann estimates and horizontal bars indicate 95% confidence intervals. Values to the left of zero indicate lower concentrations in the first group listed, whereas values to the right indicate higher concentrations.
